# Low-Hydrophilic HKUST−1/Polymer Extrudates for the PSA Separation of CO_2_/CH_4_

**DOI:** 10.3390/molecules29092069

**Published:** 2024-04-30

**Authors:** Muhamad Tahriri Rozaini, Denys I. Grekov, Mohamad Azmi Bustam, Pascaline Pré

**Affiliations:** 1 Centre of Research in Ionic Liquids, CORIL, Chemical Engineering Department, Universiti Teknologi Petronas, Bandar Seri Iskandar 32610, Perak, Malaysia or muhamad-tahriri.bin-rozaini@imt-atlantique.fr; 2 GEnie des Procédés Environnement-Agroalimentaire (GEPEA) UMR-CNRS 6144, Department of Energy Systems and Environment, IMT Atlantique, 44300 Nantes, France; denys.grekov@imt-atlantique.fr

**Keywords:** shaping, HKUST−1, MOF-polymer composite, extrusion, hydrophobic

## Abstract

HKUST−1 is an MOF adsorbent industrially produced in powder form and thus requires a post-shaping process for use as an adsorbent in fixed-bed separation processes. HKUST−1 is also sensitive to moisture, which degrades its crystalline structure. In this work, HKUST−1, in the form of crystalline powder, was extruded into pellets using a hydrophobic polymeric binder to improve its moisture stability. Thermoplastic polyurethane (TPU) was used for that purpose. The subsequent HKUST−1/TPU extrudate was then compared to HKUST−1/PLA extrudates synthesized with more hydrophilic polymer: polylactic acid (PLA), as the binder. The characterization of the composites was determined via XRD, TGA, SEM-EDS, and an N_2_ adsorption isotherm analysis. Meanwhile, the gas-separation performances of HKUST−1/TPU were investigated and compared with HKUST−1/PLA from measurements of CO_2_ and CH_4_ isotherms at three different temperatures, up to 10 bars. Lastly, the moisture stability of the composite materials was investigated via an aging analysis during storage under humid conditions. It is shown that HKUST−1’s crystalline structure was preserved in the HKUST−1/TPU extrudates. The composites also exhibited good thermal stability under 523 K, whilst their textural properties were not significantly modified compared with the pristine HKUST−1. Furthermore, both extrudates exhibited larger CO_2_ and CH_4_ adsorption capacities in comparison to the pristine HKUST−1. After three months of storage under atmospheric humid conditions, CO_2_ adsorption capacities were reduced to only 10% for HKUST−1/TPU, whereas reductions of about 25% and 54% were observed for HKUST−1/PLA and the pristine HKUST−1, respectively. This study demonstrates the interest in shaping MOF powders by extrusion using a hydrophobic thermoplastic binder to operate adsorbents with enhanced moisture stability in gas-separation columns.

## 1. Introduction

Biogas production has been increasing in recent years thanks to the implementation of multiple renewable energy policies motivated by economic and environmental benefits. From 2010 to 2019, it was estimated that global biogas production increased from 65 GW to 120 GW, of which 70% originated from Europe [1]. According to the International Energy Agency (IEA), global biogas demand is expected to reach up to 872 TWh in 2040 [2], leading to a huge potential market for this type of renewable energy. Biogas is produced from the anaerobic digestion of various organic wastes, such as sewage sludge, agricultural and crop residues, and animal dung, as well as industrial organic wastes and wastewater. Biogas consists of three main components: methane (45–70%), carbon dioxide (24–40%), and nitrogen (1–17%) [3]. Other gases that are present in biogas composition are water vapor, oxygen, hydrogen sulfide, ammonia, carbon monoxide, and traces of halogenated hydrocarbons, siloxanes, and toluene [4]. Biogas can be burned directly on-site to produce heat and electricity. However, biogas energy density is low compared to natural gas (NG) since it contains a large fraction of carbon dioxide in addition to other secondary contaminants. Therefore, biogas needs to be purified to produce biomethane, having a composition matching the specifications for injections into gas grids.

Biogas upgrading can be achieved via numerous technologies, such as water/physical/chemical scrubbing, cryogenic separation, membrane separation, pressure swing adsorption (PSA), and vacuum pressure swing adsorption (VPSA) [5,6]. In the context of this study, we are interested in the removal of CO_2_ from biogas via PSA, avoiding the use of a vacuum in the desorption step that would then be replaced with desorption in atmospheric pressure. Because vacuum desorption is mainly responsible for the high energy costs of VPSA processes, better energy performances by PSA separation are foreseen. The choice of adsorbent is one of the key factors in designing such a process.

Thermodynamic and kinetic properties of the adsorbent determine the bed working capacities and the separation performances of the whole unit in terms of methane productivity, purity, and rate of recovery. Good resistance to attrition, thermal stability over long lifetimes, and hydrophobicity are also essential to make a suitable adsorbent for such a separation. Amongst the conventional adsorbents applied for biogas upgrading in VPSA processes are zeolites and carbon molecular sieves (CMS) [7].

**Figure 1:**
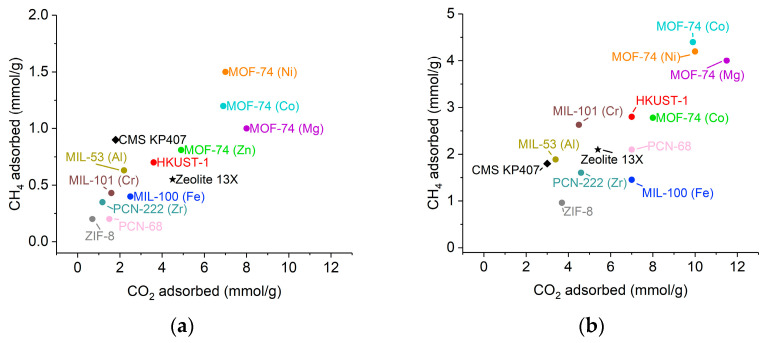
(**a**). CO_2_ and CH_4_ adsorption capacities comparison for different MOFs at 298 K and 1 bar. (**b**) CO_2_ and CH_4_ adsorption capacities comparison for different MOFs at 298 K and 5 bars.

Recently, metal–organic frameworks (MOFs) have been gaining attention for their potential application in gas separation owing to their inherent properties, such as high specific surface area, large porosity, and tunable pore size [8,9]. In the literature, MOFs have been extensively studied for their capability to separate CO_2_ from CH_4_ [10,11,12]. Amongst the materials investigated, HKUST−1 has been identified as a good candidate for CO_2_/CH_4_ separation because of its good CO_2_/CH_4_ selectivity [13,14]. The CO_2_ capture performance of HKUST−1 at low pressure is influenced by the affinity of open-metal sites with CO_2_, whereas at high pressure, it is governed by the surface area of HKUST−1 [15]. Figure 1 shows the literature comparison of CO_2_ and CH_4_ adsorption capacities of HKUST−1 with other MOFs, as well as conventional adsorbents, at 298 K for the pressure conditions of one bar and five bars, respectively [16,17,18,19,20,21,22,23,24,25,26,27,28,29]. It can be seen that CO_2_ is preferably adsorbed than CH_4_ for HKUST−1 at both pressure conditions. On the one hand, the CO_2_ adsorption capacity of HKUST−1 at both pressure conditions is better than other MOFs, such as PCN-68 and MIL-101 (Cr), as well as the conventional adsorbent CMS. On the other hand, the MOF-74 adsorbent family exhibits larger CO_2_ adsorption capacities than HKUST−1 under both pressure conditions. Similarly, at one bar, the CO_2_ adsorption capacity of zeolite 13X surpasses that of HKUST−1, though this trend is reversed at a higher pressure of five bars. Nevertheless, HKUST−1 is one of the few MOFs that are commercially available [30], making the procurement of large-scale amounts of material with consistent quality easier. However, one of the main drawbacks of using HKUST−1 as an adsorbent for CO_2_ capture from biogas is its sensitivity towards moisture, which may still be present at low concentrations in the biogas feed, even after the drying step. It is difficult to provide the exact value of leftover moisture in the biogas prior to the upgrading process, as this value may vary depending on dehumidification methods (e.g., condensation and absorption), though interested readers may refer to a notable study by Golmakani et al. [31], which summarizes the range of water dew point reduction in biogas according to different dehumidification processes. Nevertheless, the crystalline structure of HKUST is degraded when exposed to humidity, and this consequently leads to a drastic reduction in its CO_2_ adsorption capacities [32,33].

HKUST−1 could be synthesized via different synthesis routes, such as solvothermal, microwave, sonochemical, and mechanochemical synthesis [34,35,36,37,38,39,40,41,42]. HKUST−1 synthesized through these methods is usually obtained in a powder form of millimetric size, which is not convenient for use in adsorption columns because the packing of fine powder causes restrictions in the flow of gas, thus resulting in large pressure losses across the column [43]. To overcome this issue, fine crystalline adsorbent powder needs to be shaped into larger size particle forms, such as granules, tablets, or monoliths.

MOF shaping can be performed either with or without the use of a binding agent [43]. The usage of a binding agent in the shaping process may significantly modify the MOF’s intrinsic properties. In particular, it can help promote the macrostructure’s mechanical strength of the adsorbent particle and improve its chemical and thermal stability while still maintaining its intrinsic porosity and adsorption properties. For example, in a study by Cousin-Saint Remi et al. [44], ZIF-8 was shaped by a simple extrusion–crushing–sieving (ECS) approach using different binder recipes, i.e., cellulose–acetate (CA), polyvinylchloride (PVC), polyvinylformal (PVF), polyetherimide (PEI), and polystyrene (PS). The ZIF-8 composites were synthesized using 15 wt% of each binder, and it was revealed that their ethanol adsorption capacities were similar to those of the pristine ZIF-8. Furthermore, this study demonstrated that by increasing the binding agent mass fraction from 7 wt% to 30 wt%, the mechanical stability of the ZIF-8 composites was improved, and the composite made up of a PVDF binder displayed the most robust structure. In addition, it was pointed out that the composite moisture stability was dependent on the binding agent employed and that the use of PVDF as a binder yielded the most stable composite when exposed to humid conditions.

In another notable study by Hastürk et al. [45], MIL-160 (Al) and MIL-101 (Cr) were shaped via the freeze–casting method (extrusion-based method) using different hydrophilic polymeric binders, i.e., polyacrylic acid (PAA), sodium polyacrylate (PAANa), polyethylene glycol (PEG), polyvinyl alcohol (PVA) and polyvinyl pyrrolidone (PVP). This study demonstrated that the usage of a hydrophilic binder enhances the water uptake capacity of the MOFs. Both MIL-160 (Al)/MIL-101 (Cr)@polymer composites displayed an increase in water uptake capacities in the low-pressure region in comparison to the respective pristine materials.

Similarly, in our previous work [46], a pristine HKUST−1 was shaped by simple extrusion using thermoplastic polylactic acid (PLA) as the binding agent. It was shown that the HKUST−1’s crystalline structure, morphology, and textural properties, as well as CO_2_/CH_4_ adsorption capacities, were preserved in the synthesized HKUST−1/PLA composite when compared to the pristine HKUST−1. However, the moisture stability was not significantly improved in comparison to the pristine HKUST−1 [47,48]. It is, therefore, interesting to discover whether the replacement of PLA with another less-hydrophilic thermoplastic polymer as a binding agent could improve the adsorbent moisture stability.

The main objective of this research is to assess the effect of using a low-hydrophilic polymeric binder in the shaping extrusion process on the properties of an HKUST−1 adsorbent composite. For that purpose, thermoplastic polyurethane (TPU) was selected as a polymeric binding agent in order to compare the properties of HKUST−1/TPU and HKUST−1/PLA composites. Thermoplastic polyurethane (TPU) is an elastomeric polymer with a molecular configuration consisting of hard-segment and soft-segment blocks that provide properties such as high ductility, toughness, durability, flexibility, biocompatibility, and biostability [49]. The rigidity and hardness of TPU originate from the hard segment, whereas the flexibility and elastomeric properties originate from the soft segment. TPU is more hydrophobic than PLA, as demonstrated in several other studies [50,51].

To verify the successful shaping of HKUST−1 with TPU, structural and textural properties, as well as CO_2_ and CH_4_ adsorption equilibrium data for HKUST−1/TPU, were measured and subsequently compared with pristine HKUST−1 and HKUST−1/PLA composites synthesized from the same extrusion process as reported in [46]; however, we used thermoplastic polylactic acid (PLA) as the binding agent. The hydrophilicity of the two polymers was quantified in this study by conducting surface wettability tests (Figure S1). The effect of methanol washing on the textural properties and adsorption capacities of HKUST−1 powder was assessed to interpret differences observed between the composites and pristine HKUST−1. Finally, the stability of the samples under humid conditions was analyzed to assess the impact of the polymer’s hydrophobicity on the moisture stability of the composites.

## 2. Results and Discussion

### 2.1. Sample Characterization

**Figure 2:**
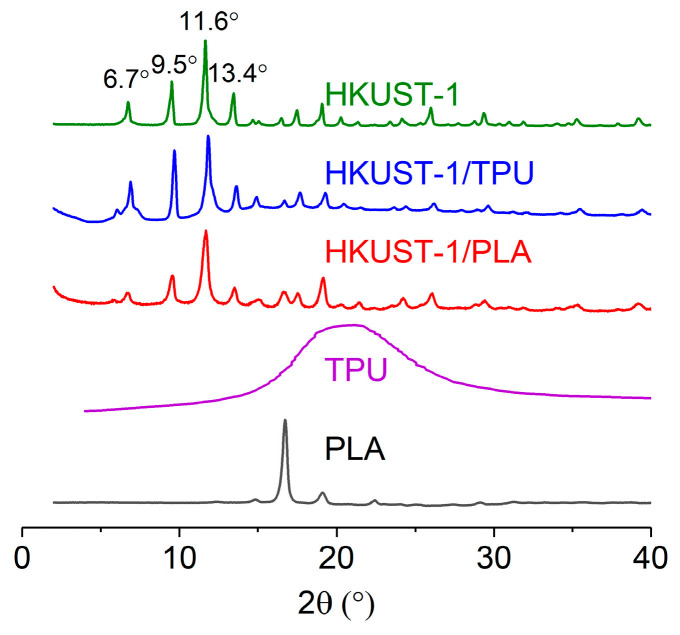
XRD of TPU, PLA, pristine HKUST−1 and its composites.

The structural analysis of the samples was investigated through an XRD analysis. Figure 2 illustrates XRD peaks observed for HKUST−1/TPU, HKUST−1/PLA, pristine HKUST−1, TPU, and PLA. Pure TPU exhibited a characteristic broad peak around 20° due to the diffraction from the (110) planes of the TPU soft segments [52,53], whereas pure PLA exhibited a strong peak at 16.8° due to diffraction from (110) and/or (200) planes [54]. Additionally, pristine HKUST−1 exhibited characteristic peaks at 6.7°, 9.5°, 11.6°, and 13.4°, which can be attributed to the (200), (220), (222), and (400) crystal planes of HKUST−1 [46].

The XRD pattern for HKUST−1/TPU closely resembled the one for HKUST−1/PLA, where peaks associated with pristine HKUST−1 were clearly present in both composites, whereas peaks associated with pure TPU or PLA were indistinguishable in the HKUST−1/TPU and HKUST−1/PLA samples due to their low loading in the composites. Nevertheless, the XRD analysis confirms the presence of HKUST−1 particles in both composites after the shaping process.

**Figure 3:**
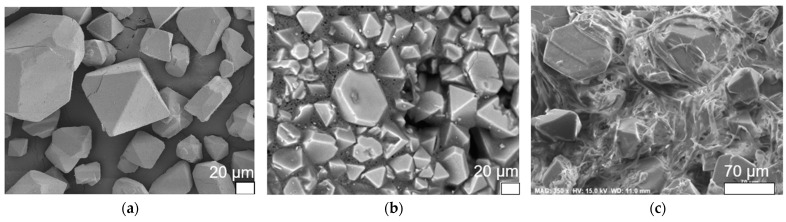
SEM image of (**a**) pristine HKUST−1. (**b**) HKUST−1/PLA. Reproduced from [46]. (**c**) HKUST−1/TPU.

**Figure 4:**
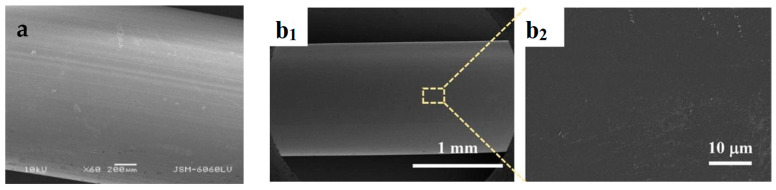
(**a**) SEM image of neat PLA filament. Image reproduced from [55]. (**b_1_**,**b_2_**) SEM image of neat TPU filament at different magnification. Images reproduced with permission from [56].

Scanning electron microscopy (SEM) imaging was used to characterize the surface morphology of the adsorbent materials. Figure 3 compares the morphology observed between HKUST−1/TPU, HKUST−1/PLA, and the pristine HKUST−1. Most of the pristine HKUST−1 particles exhibited an octahedron shape, though there were also large particles of an irregular shape. Morphologies of the surface of HKUST−1/TPU and HKUST−1/PLA show that the particles of HKUST−1 were not totally encapsulated by the polymeric binder after shaping. Furthermore, it can be observed that particles in the HKUST−1/TPU composite were held together by fibrous TPU polymers, whereas particles in the HKUST−1/PLA composite were surrounded by amorphous PLA. It is likely that the formation of the fiber grid observed in HKUST−1/TPU occurred during the synthesis process of the composite, as typical commercial filaments of TPU and PLA exhibited a smooth and dense surface (Figure 4). The obtained SEM imaging of both composites demonstrates that the shaping process, using a 10% mass of either one of the polymeric binders, yielded a composite in which HKUST−1 particles remained accessible for gas adsorption.

**Figure 5:**
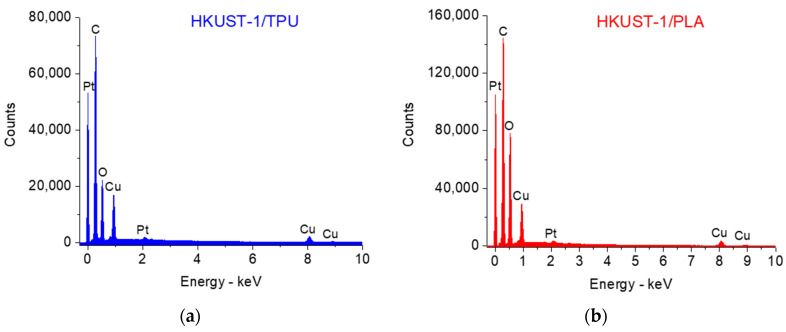
(**a**) EDS spectra for HKUST−1/TPU. (**b**) EDS spectra for HKUST−1/PLA.

The EDS spectra of HKUST−1/TPU and HKUST−1/PLA are presented in Figure 5a and Figure 5b, respectively. Qualitatively, the peaks corresponding to the expected main elements in the composites are evident, namely, C, O, and Cu. C and O elements were present in both HKUST−1 and the polymeric binder, whereas the Cu element was only present in the pristine HKUST−1. It should be noted that the difference in an element’s relative content in HKUST−1/TPU and HKUST−1/PLA was not exploited as the samples that were used for the analysis were not flat, which rendered the quantitative analysis of EDS unreliable. Nevertheless, the presence of element peaks in the composite was similar to the EDS spectra of the pristine HKUST−1 powder reported in other studies [57,58], which is indicative of the preservation of the HKUST−1 crystals in both composites.

**Figure 6:**
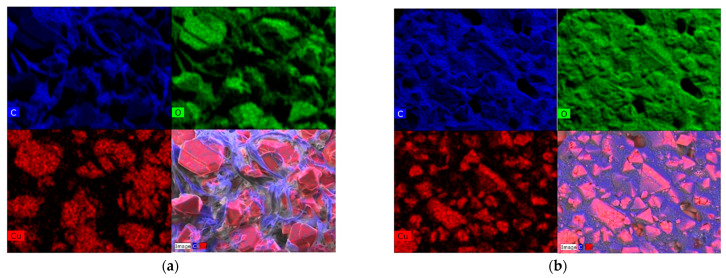
(**a**) EDS mapping for HKUST−1/TPU. (**b**) EDS mapping for HKUST−1/PLA.

Figure 6 displays elemental mapping for HKUST−1/TPU and HKUST−1/PLA. The mapping reveals a uniform distribution of C (represented by blue color) and O (represented by green color) elements over the surface of the composites because these elements were present in both the MOF and binder. However, the Cu element (represented by red color) had a more heterogeneous distribution as this element was only present in HKUST−1. By combining the mapping of the Cu and C elements together, we could observe that there was a clear separation between the Cu element in HKUST−1 and the C element in the polymeric binder of both composites. This separation, clearly observed from the elemental mapping, is a good indication that no degradation reaction occurred between HKUST−1 and the polymeric binders (TPU or PLA) during shaping. It should be noted here that there is a grayish section in the elemental mapping that corresponds to the area where the EDS analysis was unable to detect the elements due to shadowing.

**Figure 7:**
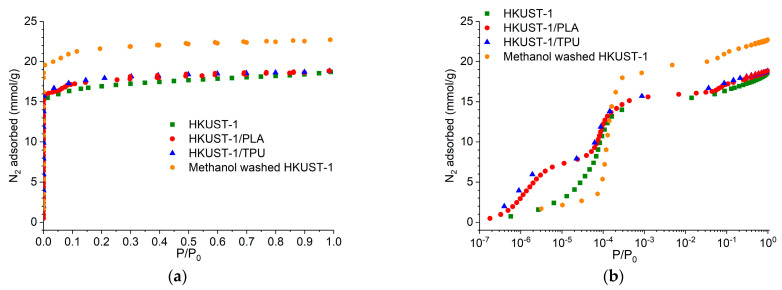
N_2_ adsorption isotherm plot for HKUST−1/TPU, HKUST−1/PLA, pristine HKUST−1 and methanol-washed HKUST−1 in (**a**) linear scale and in (**b**) log scale. Data for HKUST−1/PLA and HKUST−1 was taken from previous work [46].

Nitrogen physisorption isotherms are shown in Figure 7 on both linear and logarithmic scales. All isotherms featured a type I shape relevant to highly microporous solids [59]. The N_2_ adsorption capacities of HKUST−1/TPU and HKUST−1/PLA were slightly larger than the pristine HKUST−1’s, which signifies that the pristine HKUST−1 particles in both composites remained accessible to gas adsorption, as shown by SEM imaging. In addition to that, it can be seen that nitrogen filling in the lowest pressure range (P/P_0_ < 10^−4^) was different for both composites when compared to the pristine HKUST−1 powder. This indicates some changes related to the accessibility of the primary adsorption sites located in the microporous domain after shaping. Furthermore, the N_2_ adsorption isotherm of pure TPU and pure PLA (Figure S2, Supporting Information) reveals that both polymeric binders absorbed a very negligible amount of N_2_ in comparison to pristine HKUST−1 and its composites, which signifies that both TPU and PLA did not contribute to the slightly larger N_2_ adsorption capacity denoted for the two composites. Meanwhile, when pristine HKUST−1 was washed with methanol, an increase in the N_2_ adsorption capacity could be observed. Therefore, the increase in the N_2_ adsorption capacity for both composites could be explained by the effect of “washing” with methanol during the shaping process.

Table 1 lists the BET surface area and the pore volume data derived from 77K-N_2_ isotherms for HKUST−1/TPU, HKUST−1/PLA, pristine HKUST−1, and methanol-washed HKUST−1. As expected, the micropore volumes of both HKUST−1/TPU and HKUST−1/PLA were slightly higher than pristine HKUST−1’s, matching the changes observed in the low-pressure region of the N_2_ isotherms. The computed BET surface areas of HKUST−1/TPU and HKUST−1/PLA appeared a bit larger than that of pristine HKUST−1. Once again, these trends could be explained by the “washing” of the composites with methanol, as methanol-washed HKUST−1 recorded an increase in the BET surface area and pore volume in comparison to the pristine HKUST−1. Additionally, when pristine HKUST−1 was washed with either DMF or chloroform, an increase in the BET surface area and/or pore volume could also be observed (Figure S3 and Table S2, Supporting Information), which may have resulted from residual reagents present in the pores washed away by the solvents.

### 2.2. Thermal and Mechanical Stability

**Figure 8:**
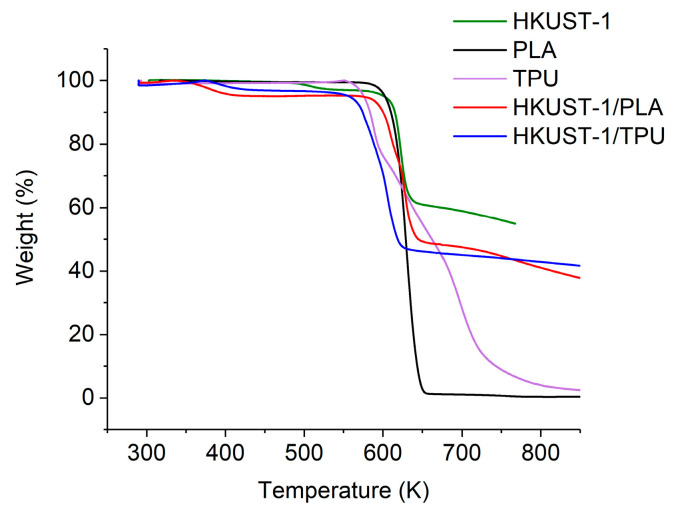
TGA profile for TPU, PLA, pristine HKUST−1 and its composites. Data for HKUST−1 and PLA was taken from previous work [46].

The TGA profiles for pristine HKUST−1 and its composites, as well as for the neat PLA and TPU, are shown in Figure 8. The pristine HKUST−1 was thermally stable up to 593 K, whereas PLA started to degrade at 613 K. Meanwhile, the thermal degradation behavior of TPU can be divided into two stages; the first stage of decomposition occurred between 553 K and 613 K, while the second stage occurred between 663 K and 733 K. The reason for the two-stage degradation was related to the decomposition behavior of urethane bonds in the hard segments and polyol groups in the soft segments of the TPU polymer [60,61].

The thermal degradation of HKUST−1/TPU and HKUST−1/PLA proceeded in two steps; a first mass loss occurred between 350 K–410 K, which consisted of a 3% and 5% weight loss for HKUST−1/TPU and HKUST−1/PLA, respectively, which was associated with the loss of leftover solvent molecules (chloroform, DMF or methanol) that remained trapped inside the pores; the second mass loss started around 560 K and 590 K for HKUST−1/TPU and HKUST−1/PLA, respectively, which can be attributed to the start of the framework and polymeric binder degradation. Interestingly, the mass loss due to trapped solvent molecules is significantly less for HKUST−1/TPU in comparison to HKUST−1/PLA, which could be due to the fibrous nature of TPU in the composite, as seen in the SEM imaging, limiting the entrapment of the solvent after the shaping process. Furthermore, the decomposition behavior of both the hard and soft segments of TPU was no longer distinguishable in the HKUST−1/TPU composite. It could be observed that both the HKUST−1/TPU and HKUST−1/PLA composites displayed a lower degradation temperature than pristine HKUST−1 and their respective pure polymeric binders. This could be attributed to the presence of metal from MOFs in the composite that may have acted as a catalyst for polymer degradation, as concluded in several other studies [54,62,63].

The attrition values of the samples were investigated to determine their mechanical stabilities. An adsorbent with a high attrition value could lead to problems when operating the gas-separation unit, such as bed clogging or an increase in pressure drops inside the adsorption column. Table 2 compares the attrition values measured for HKUST−1/TPU and HKUST−1/PLA with commercial adsorbents and MOF extrudates from other studies. These values were determined using the same attrition test standard according to the D4058-96 ASTM norm [64]. The attrition value of HKUST−1/TPU was only slightly lower in comparison to the one of HKUST−1/PLA, but both samples possessed comparable mechanical stability compared with other commercial adsorbents, as reported in [65]. This shows that TPU and PLA, used at a low loading rate as binding agents, are suitable for making HKUST−1 adsorbent composites with good mechanical resistance to attrition. To improve the mechanical stability of HKUST−1 composites, it could be possible to increase the concentration of the binder, but there is a risk of decreasing the gas-separation performance of the composites because of pore blockages, as observed for the HKUST−1/PLA produced at a 20 wt% binder loading (Figure S4).

### 2.3. CO_2_ and CH_4_ Adsorption Measurements

**Figure 9:**
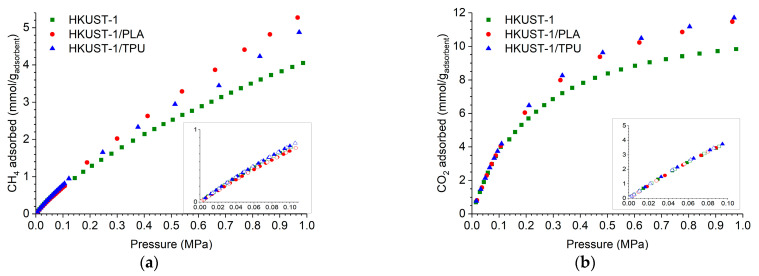
Gravimetric adsorption isotherms of (**a**) CH_4_ and (**b**) CO_2_ on HKUST−1 and its composites at 298 K. The inset displays the adsorption isotherm at equilibrium pressure up to 1 bar. Data for HKUST−1 and HKUST−1/PLA was taken from previous work [46].

Methane and carbon dioxide adsorption isotherms (expressed as mmol/g of the adsorbent), which were measured at 298 K for HKUST−1/TPU, HKUST−1/PLA, and pristine HKUST−1, are presented in Figure 9. In a pressure range of less than one bar, both CO_2_ and CH_4_ adsorption capacities were similar for all the samples and appeared to be linear with the equilibrium pressure. In a pressure range between 1 and 10 bars, the shape of the CO_2_ isotherms for all the samples was no longer linear with the equilibrium pressure. The CO_2_ adsorption capacities tended to reach a plateau, which was, however, not observable in the tested equilibrium pressure conditions. The CH_4_ adsorption isotherms of all the materials in that same pressure range remained, however, almost linear with the equilibrium pressure.

The CO_2_ and CH_4_ adsorption capacities of the HKUST−1/TPU composite were similar to HKUST−1/PLA and pristine HKUST−1 at equilibrium pressures of less than one bar. However, as the equilibrium pressure increased, both composites exhibited larger CO_2_ and CH_4_ adsorption capacities than the pristine HKUST−1, which may be attributed to their larger BET surface areas and micropore volumes. The CO_2_ adsorption isotherm of HKUST−1/TPU closely resembled that of HKUST−1/PLA, which suggests that TPU as a binding agent does not have a different effect from PLA on CO_2_ adsorption capacities. Similarly, the CH_4_ adsorption capacities of HKUST−1/TPU were comparable to those of HKUST−1/PLA in the upper range of the equilibrium pressure.

**Figure 10:**
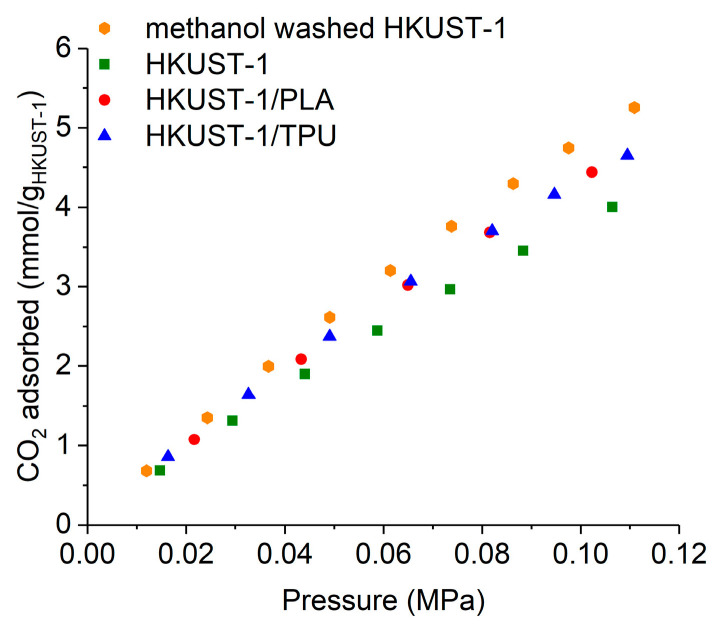
Normalized CO_2_ adsorption isotherms compared for pristine HKUST−1, methanol-washed HKUST−1, HKUST−1/PLA and HKUST−1/TPU.

Previous N_2_ adsorption isotherm measurements reveal that both composites exhibited higher BET surface areas and pore volumes than the pristine HKUST−1, which may be explained by the washing effect with methanol. Therefore, it is interesting to compare the adsorption performances of the active HKUST−1 particles present in both composites with both pristine and methanol-washed HKUST−1 powders, as displayed in Figure 10. From the normalized CO_2_ adsorption capacities, it is shown that at 298 K, HKUST−1 particles in both composites had nearly equal adsorption capacities. In addition, the embedded particles exhibited higher CO_2_ adsorption capacities than the pristine HKUST−1 but slightly lower capacities (by around 10%) than the methanol-washed HKUST−1 powder. This could be attributed to partial pore blockage by the thermoplastic binder.

### 2.4. Effect of Temperature on Adsorption Isotherms

**Figure 11:**
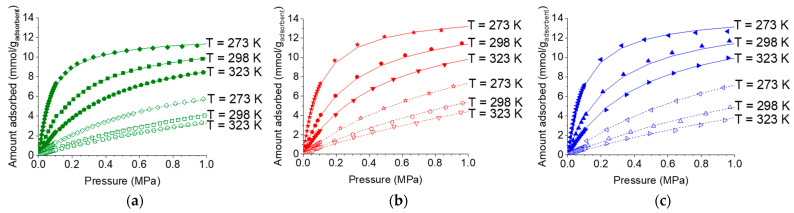
Gravimetric adsorption isotherms of CH_4_ and CO_2_ at different temperatures for (**a**) pristine HKUST−1, (**b**) HKUST−1/PLA, and (**c**) HKUST−1/TPU. Continuous line represents dual-site Langmuir modeling, whereas filled and unfilled symbols represent CO_2_ and CH_4_ adsorption, respectively. Isotherm data for pristine HKUST−1 and HKUST−1/PLA were taken from previous work [46].

Figure 11 shows the CO_2_ and CH_4_ adsorption isotherms of all the materials measured at 273 K, 298 K, and 303 K. Unsurprisingly, it can be observed that an increase in temperature resulted in a decrease in both CO_2_ and CH_4_ adsorption capacities. Furthermore, both composites had comparable CO_2_ and CH_4_ adsorption capacities at any given temperature.

### 2.5. Temperature Dependent Isotherm Modeling and Isosteric Heat of Adsorption

The determination of the isosteric heat of adsorption Q_ST_ requires a linear regression of the equilibrium data. Thus, to ensure the accuracy of the calculation, an appropriate thermodynamic model was chosen, ensuring good fitting with the experimental isotherm data in the whole temperature range. The dual-site Langmuir model was applied in this study to describe the pure component isotherm data of all the materials, taking into account the influence of temperature, according to the following equations:

(1) $$q_{\mathrm{eq},i}=q_{s1,i}\cdot \frac{K_{1,i}\cdot P_{i}}{1+\left(K_{1,i}\cdot P_{i}\right)}+q_{s2,i}\cdot \frac{K_{2,i}\cdot P_{i}}{1+\left(K_{2,i}\cdot P_{i}\right)}\;K_{1,i}=k_{a1,i}e^{k_{a2,i}\cdot \left(\frac{1}{T}-\frac{1}{T_{\mathrm{ref}}}\right)}\;K_{2,i}=k_{b1,i}e^{k_{b2,i}\cdot \left(\frac{1}{T}-\frac{1}{T_{\mathrm{ref}}}\right)}$$

q_s1,i_ and q_s2,i_ were the saturation capacities of component i in the gas mixture on site 1 and site 2, respectively; K_1,I_ and K_2,i_ were the equilibrium constants of component i in the gas mixture on site 1 and site 2; k_a1,i_ and k_b1,i_ were the pre-exponential constants for the temperature dependence of K_1,i_ and K_2,i_, respectively; k_a2,i_ and k_b2,i_ were the exponential terms for the temperature dependence of K_1,i_ and K_2,i_, respectively; P_i_ was the pressure of gas component i in equilibrium with the adsorbed phase; q_eq,i_ was the amount adsorbed of component i at equilibrium; T_ref_ was the reference temperature. The goodness of fitting was quantified considering the determination coefficient values R^2^, as presented in Table S1 (Supporting Information).

The application of the dual-site Langmuir model to describe the experimental CO_2_ adsorption isotherms up to 10 bars reveals that the monolayer saturation capacity of adsorption site 1, q_s1_, was higher than the monolayer saturation capacity of adsorption site 2, q_s2_, for all the samples. It is likely that q_s1_ corresponds to the saturation of the polarized adsorption sites located near the open-metal sites of HKUST−1, whereas qs_2_ describes the monolayer saturation capacity over sites near the ligand, as proposed in [13,67]. In the case of CH_4_, the values of q_s1_ derived from the dual-site Langmuir model were found superior to q_s2_ for all the samples. However, according to the literature [13,68], the preferential adsorption sites of CH_4_ on HKUST−1 were located in the octahedral cages of the ligand, whereas adsorption close to the metal sites was less favored due to their lesser accessibility. Therefore, q_s1_ most likely represents the monolayer CH_4_ saturation capacity near the ligand, whereas q_s2_ describes the adsorption capacity of CH_4_ for the sites near the open-metal sites of the HKUST−1 crystalline structure.

**Figure 12:**
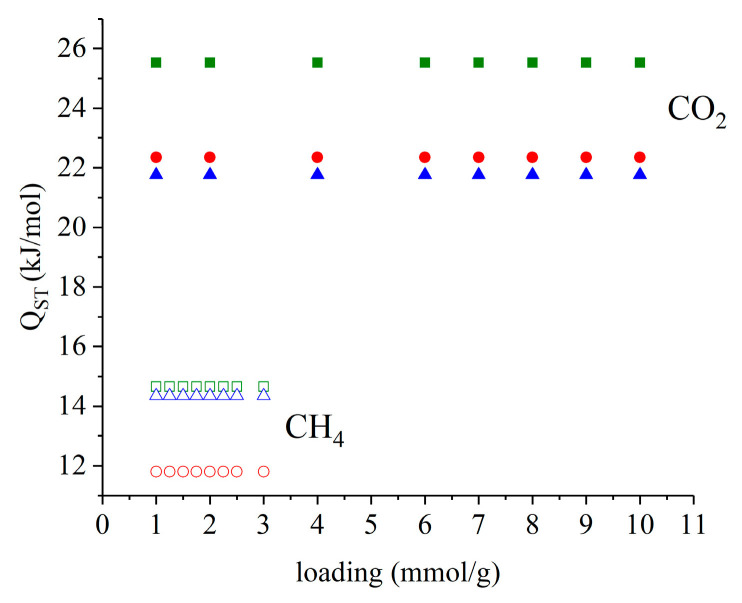
CO_2_/CH_4_ isosteric heats of adsorption for HKUST−1/TPU (blue triangle), HKUST−1/PLA (red circle) and pristine HKUST−1 (green square). Data for pristine HKUST−1 and HKUST−1/PLA were taken from previous work [46].

Once the fitting of the isotherm data with the dual-site Langmuir model was established, the value of Q_ST_ could be determined from the linear regression of the equilibrium data. Figure 12 presents the CO_2_/CH_4_ isosteric heats of adsorption calculated from the Clausius–Clapeyron equation at different loadings of the adsorbed gas for pristine HKUST−1, HKUST−1/TPU, and HKUST−1/PLA. It could be observed that the CO_2_ and CH_4_ heats of adsorption were constant for all the samples as the loading of the adsorbed gas increased. Both HKUST/TPU and HKUST−1/PLA possessed lower CO_2_ adsorption heat than pristine HKUST−1. It could be proposed that the presence of TPU in the composite lessened the interaction intensity between the CO_2_ molecules and adsorption sites on HKUST−1. Interestingly, the HKUST−1/PLA composite exhibited a slightly lower CH_4_ adsorption heat compared to pristine HKUST−1, whereas HKUST−1/TPU and pristine HKUST−1 showed similar CH_4_ adsorption heats. This would mean that the presence of TPU in the adsorbent composite did not influence the interactions between CH_4_ and adsorption sites of HKUST−1, contrary to CO_2_.

### 2.6. Prediction of CO_2_/CH_4_ Co-Adsorption Isotherm and IAST Selectivites

**Figure 13:**
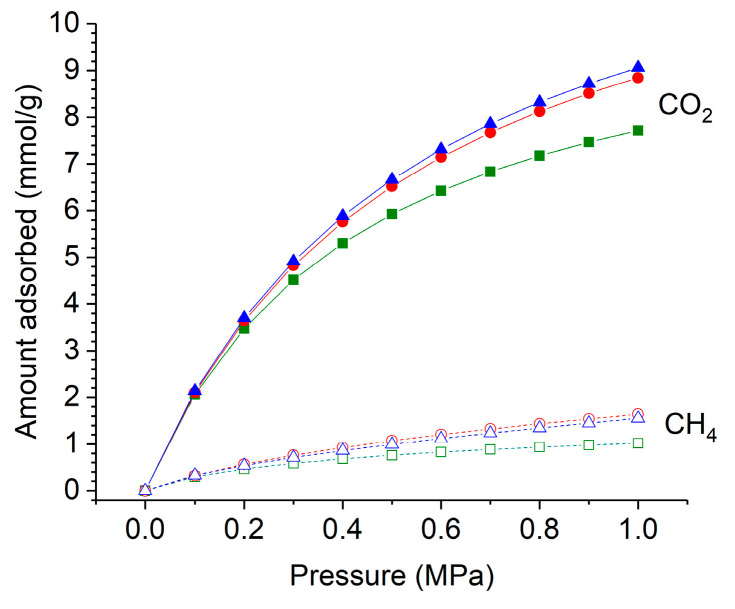
IAST-predicted co-adsorption isotherms for equimolar CO_2_/CH_4_ mixtures on HKUST−1 (green square), HKUST−1/PLA (red circle) and HKUST−1/TPU (blue triangle) at 298 K as a function of total bulk pressure. Data for pristine HKUST−1 and HKUST−1/PLA were taken from previous study [46].

By using the dual-site Langmuir isotherm model for the fitting of CO_2_ and CH_4_ equilibrium data, the co-adsorption isotherms of an equimolar mixture of CO_2_/CH_4_, representative as an average to the composition of an inlet biogas stream [4], were predicted using IAST for all the materials. Figure 13 describes the predicted co-adsorption isotherms of HKUST−1/TPU, HKUST−1/PLA, and pristine HKUST−1 at 298 K, up to 10 bars. Both HKUST−1/TPU and HKUST−1/PLA composites were predicted to have larger CO_2_ and CH_4_ co-adsorption capacities compared with pristine HKUST−1. Furthermore, for all the materials, CO_2_ was more preferably adsorbed than CH_4_ in these conditions.

The adsorbent working capacity is one of the key characteristics in designing a PSA process. The working capacity is defined as the difference between the adsorbed quantities determined under equilibrium at the operating adsorption and purging pressures, respectively. Assuming a PSA process of biogas upgrading, where the adsorption pressure and purging pressure are 10 bars and 1 bar, respectively, the predicted CO_2_ and CH_4_ working capacities between HKUST−1/TPU and HKUST−1/TPU are very similar to each other. This suggests that the usage of either one or the other composite will result in similar gas-separation performances.

**Figure 14:**
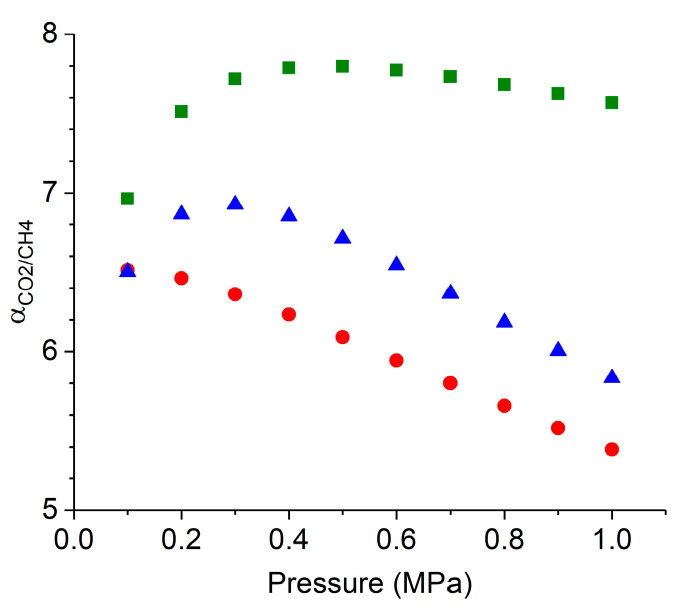
IAST-predicted selectivities for equimolar CO_2_/CH_4_ mixtures on pristine HKUST−1 (green square), HKUST−1/PLA (red circle) and HKUST−1/TPU (blue triangle) at 298 K as a function of total bulk pressure. Data for pristine HKUST−1 and HKUST−1/PLA were taken from previous study [46].

All the samples exhibited a better affinity towards CO_2_ than CH_4_. For all the materials, the ratio of equilibrium capacities of pure components was around 2. The prediction of co-adsorption isotherms via IAST allowed for the determination of the equilibrium selectivities of an equimolar binary mixture. Figure 14 presents the CO_2_/CH_4_ co-adsorption selectivities as a function of the equilibrium pressure at 298 K for HKUST−1/TPU, HKUST−1/PLA, and the pristine HKUST−1, respectively. It could be observed that CO_2_ was more preferentially adsorbed than CH_4_ for all the materials. Additionally, pristine HKUST−1 had the highest selectivity value in the whole equilibrium pressure range, followed by HKUST−1/TPU and HKUST−1/PLA. Furthermore, as the equilibrium pressure was greater than 3 bars, the selectivities of HKUST−1/TPU and HKUST−1/PLA were noticeably diminished, even if the variations remained small, whereas the selectivity of the pristine HKUST−1 did not vary significantly. This could be explained by the larger CH_4_ adsorption capacities of the composites, which lowered the separation selectivity despite their larger CO_2_ adsorption capacities. When comparing both the composites, HKUST−1/TPU was shown to be slightly more selective towards CO_2_ than HKUST−1/PLA, as the former had a slightly lower CH_4_ adsorption capacity than the latter.

### 2.7. Aging along Exposure in Humid Conditions

A water contact angle analysis was conducted on both polymers (Figure S1) to determine their surface wettability and, thus, the hydrophobicity of the material. The average water contact angles for TPU and PLA are presented in Table 3. Based on the water contact angle value of each polymer, it can be concluded that TPU was more hydrophobic than PLA. Therefore, the presence of TPU as a binding agent instead of PLA could contribute to the improvement of the moisture stability of the HKUST−1 composite, which would corroborate the findings of other studies [69,70,71].

**Figure 15:**
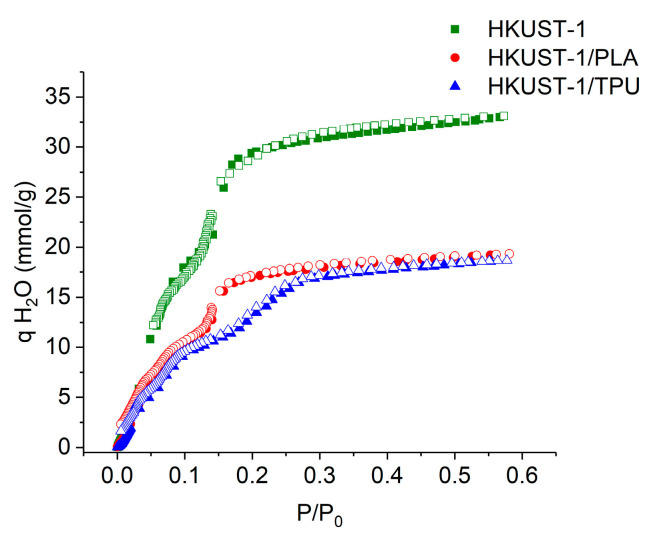
H_2_O adsorption isotherm plot for HKUST−1/TPU, HKUST−1/PLA and pristine HKUST−1 at 298 K.

HKUST−1 is known as a MOF that is sensitive to water as the open-metal sites of HKUST−1 have a high affinity with water molecules, which results in the formation of Cu-O bonds and thus leads to the disintegration of the HKUST−1 framework [72,73]. Figure 15 displays the H_2_O adsorption isotherm for HKUST−1/TPU, HKUST−1/PLA, and pristine HKUST−1 at 298 K. As expected, the pristine HKUST−1 was able to adsorb a larger quantity of water than the extruded composites when the relative humidity (RH) increased. Furthermore, the accessible pores of the pristine HKUST−1 sample started to be completely filled with water at P/P_0_ ≈ 0.17. Meanwhile, the presence of either TPU or PLA in the composite had an effect on lowering the quantity of the adsorbed water molecules as the relative pressure increased. It is possible that the polymeric binder partially hindered the access of the water molecules to certain open-metal sites in HKUST−1. Interestingly, it can be noticed that the accessible pores in HKUST−1/PLA started to be filled with water at a similar relative pressure as pristine HKUST−1, while this was not true in HKUST−1/TPU as the accessible pores only started to be filled at P/P_0_ ≈ 0.27, which could be a result of the hydrophobic nature of TPU reducing the interaction intensity of the MOF open-metal sites with the water.

**Figure 16:**
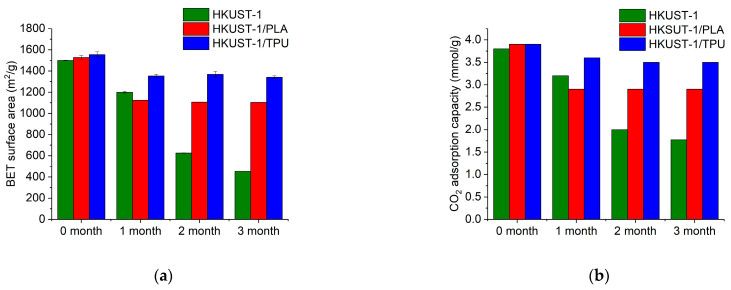
Variation of (**a**) BET surface area and (**b**) CO_2_ adsorption capacity of pristine HKUST−1, HKUST−1/PLA and HKUST−1/TPU extrudates stored in humid conditions for 3 months.

The composite and pristine samples were stored inside a humid environment (RH = 40 ± 5%) for a duration of 3 months, and the samples were re-characterized during this period to investigate the aging of the materials. The value of air RH was sufficiently high to ensure that the pores of all the samples could be filled with humid air based on the previous H_2_O isotherm plot for HKUST−1 and its composites. Figure 16 presents the variation in the BET surface areas as well as those of the CO_2_ adsorption capacities at one bar and 298 K for HKUST−1/TPU, HKUST−1/PLA, and the pristine HKUST−1 after 3 months of storage in a humid environment. As expected, pristine HKUST−1 showed degradation of both the BET surface and CO_2_ adsorption capacities after 3 months of storage in a humid environment. Meanwhile, HKUST−1/PLA had a similar degradation pattern as the pristine HKUST−1 during the first month of storage, but the degradation seemed to stabilize after 1 month. Interestingly, HKUST−1/TPU exhibited less sensitivity to degradation under humid exposure than HKUST−1/PLA during the first month, which, moreover, seemed to be halted after 1 month of storage. We hypothesized that since TPU is more hydrophobic than PLA, it would contribute to a slower degradation of HKUST−1 in the composite. Additionally, both polymeric binders may have hindered some access to the MOF open-metal sites, which are the preferential adsorption sites for water molecules, which could also explain the stabilization of the degradation for both composites after 1 month of storage.

## 3. Materials and Methods

### 3.1. Materials

HKUST−1 (also known as Cu-BTC) is a MOF also commercially known as Basolite@C300, a trademark of BASF (Beaumont, TX USA) SE. This material, denoted as the pristine HKUST−1, was supplied in powder form by Sigma Aldrich (Saint Louis, MO, USA). Its specific surface area, as specified by the supplier, is in the range of 1500–2100 m^2^/g, and its bulk density is 0.35 g/cm^3^. In addition, the particles of Basolite@C300 have an average size of 15.96 μm. Apart from that, another sample denoted as methanol-washed HHKUST−1 was also prepared by treating/washing the pristine HKUST−1 with methanol. TPU filament (Python Flex) was purchased from FormFutura (Amsterdam, The Netherlands). The melting temperature of the TPU is in the range of 493 K–523 K, according to the supplier.

### 3.2. Synthesis of HKUST−1/TPU Composite

Prior to synthesis, pristine HKUST−1 powder and TPU, as received, were degassed under a vacuum at 473 K and 383 K, respectively.

**Figure 17:**
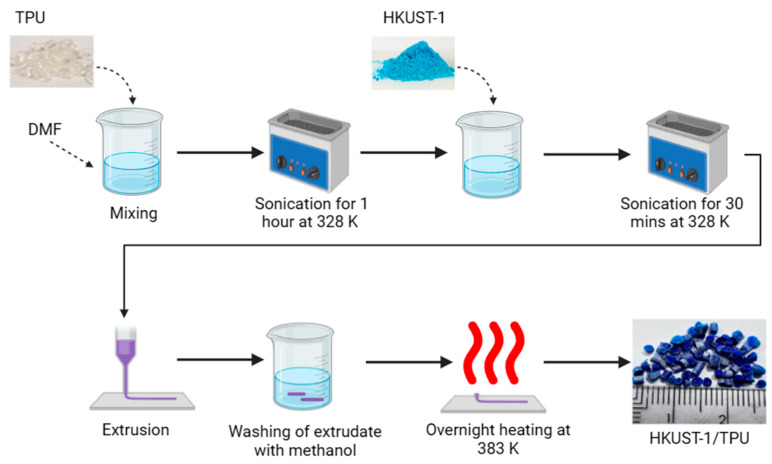
Schematic figure of the shaping process of HKUST−1/TPU composite shaping. Dashed line arrow signifies addition of material inside a recipient whereas continuous line arrow signifies the order of the synthesis step.

The shaping extrusion process applied to produce HKUST−1/TPU was the same as the one employed to produce the HKUST−1/PLA composite and is described in detail in [46]. Briefly, 0.1 g of TPU was dissolved inside 1 mL of solvent (DMF) using an ultrasonic bath at 328 K for the duration of 1 h. Once TPU was dissolved, 0.9 g of pristine HKUST−1 powder was gradually added to the solution, and further sonification was performed for 30 min to obtain a homogenous mixture that contained 10 wt% binder content. The HKUST−1/TPU suspension was then inserted into a 5 mL DB syringe, followed by its extrusion. Next, the extruded HKUST−1/TPU was briefly washed with methanol to promote an exchange with DMF. Methanol, in having a lower boiling point than DMF, promoted a thorough drying of the HKUST−1/TPU composite. The drying step was performed overnight at 383 K and under vacuum to remove the leftover solvent molecules (DMF or methanol). The dried HKUST−1/TPU composite was then cut using scissors into small cylinders of about 1–2 mm in length. Figure 17 illustrates the synthesis process of HKUST−1/TPU.

### 3.3. Scanning Electron Microscopy

A JEOL (Peabody, MA, USA) JSM 7600F high-resolution scanning electron microscope was used to collect images of the samples using a 15 kV electron beam, and it was equipped with both backscattered electron (BSE) and secondary electron (SE) detectors. The material samples were deposited on double-sided carbon tape and metalized with Pt. BSE imaging was used to carry out an energy-dispersive X-ray spectroscopy (EDS) element mapping of the samples.

### 3.4. Powder X-ray Diffraction (XRD)

A Bruker D8 Advance (Billerica, MA, USA) diffractometer equipped with a copper anode (λ = 1.5406 Å) was used to collect XRD patterns. Data were collected in the 2θ range from 5 to 50°, with a step of 0.02° and a scan speed of 1°/min.

### 3.5. Thermogravimetric Analysis

Thermogravimetric analysis (TGA) measurements were performed using a Setaram (Caluire, France) SETSYS Evolution thermogravimetric analyzer. A total of 5 mg of samples was heated in platinum crucibles from an ambient temperature of up to 1000 K under 20 mL/L of dry N_2_ flow at a heating rate of 10 °C/min. The blank was subtracted to correct the TG signal. Prior to analysis, both HKUST−1 composite sample were kept in a desiccator for a prolonged time, more than 1 month, and heated at 383 K overnight under vacuum.

### 3.6. Characterization of Textural Properties

The material-specific surface area was determined using 77 K-N_2_ adsorption isotherm data, assuming the BET theory. The total porous volume was determined from experimental N_2_ sorption data at P/P_0_ = 0.98. The micropore volume was derived by applying the t-plot model to the N_2_ adsorption isotherms.

The N_2_ adsorption equilibrium data were obtained using a 3 Flex manometric adsorption analyzer from Micromeritics (Norcross, GA, USA). The instrument was equipped with pressure transducers, allowing measurements in the domain of relative pressure (P/P_0_) ranging between 10^−7^ and 1, with a 0.15% precision of the absolute pressure reading. The selected pressure range for the BET surface area calculation was chosen in the domain of 10^−7^ ≤ P/P_0_ ≤ 10^−2^ to respect the four consistency criteria of the BET equation, as suggested by Rouquerol et al. [74]. Prior to isotherm measurement, the sample was degassed at 383 K under vacuum for at least 72 h. The void volume of the cell containing the sample was evaluated from helium expansion measured at an ambient temperature and at 77 K.

### 3.7. Attrition Test

The mechanical stability of the samples was characterized through the attrition test according to the standard D4058-96 ASTM norm. Briefly, 0.2 g mass of material was introduced into a glass vial and was rolled at a frequency of 60 revolutions per minute (rpm) for 30 min. Afterward, the sample was passed through a 500 μm sieve to recover fine particles. The attrition percentage was calculated as follows.

(2) $$\mathrm{Attrition}\;(\%)=\frac{\mathrm{initial}\;\mathrm{mass}-\mathrm{recovered}\;\mathrm{mass}\;\mathrm{above}\;500\;\mu m}{\mathrm{initial}\;\mathrm{mass}}\;\times \;100$$

### 3.8. CO_2_ and CH_4_ Adsorption Isotherms

CO_2_ and CH_4_ adsorption isotherms were measured using two manometric pieces of equipment. The isotherm data in the pressure range from 1 to 10 bars were collected using the Pressure-Composition Thermodynamics (PCT)-Pro (manometric equipment from SETARAM, Caluire, France). In the low-pressure range, less than 1 bar, the isotherm data were obtained with the 3 Flex apparatus from Micromeritics. In both the low- and high-pressure ranges, adsorption isotherms data were collected at three temperatures: 273 K, 298 K, and 323 K. Prior to each measurement, the samples were degassed under a dynamic vacuum at 383 K for 12 h, and the sample holder dead volume was measured from helium expansion.

### 3.9. Isosteric Heat of Adsorption

The heat of the adsorption of the components of a gas mixture is a key thermodynamic variable for the design of practical gas-separation processes such as pressure swing and thermal swing adsorption [75,76]. Knowledge of the heat of adsorption helps to determine the extent of adsorbent temperature changes within the adsorbent bed during the adsorption and regeneration processes, which ultimately govern the gas-separation performance. Apart from that, it also allows for the quantification of the adsorbent–adsorbate interaction intensity. The isosteric heats of the adsorption were derived from the application of the Clausius–Clapeyron equation:

(3) $$\ln\frac{P_{2}}{P_{1}}=\frac{Q_{\mathrm{ST}}}{R}\;\times \;(\frac{1}{T_{1}}-\frac{1}{T_{2}})$$

where Q_ST_ was the isosteric heat of adsorption, T was the temperature, and R was the universal gas constant with a value of 8.314 J/mol K. The values of Q_ST_ were determined for different gas loadings, regressing the isotherm data measured at three different temperatures.

### 3.10. Ideal Adsorption Solution Theory (IAST)

The Ideal Adsorption Solution Theory, developed by Myers and Prausnitz [77], is a thermodynamic model enabling the prediction of co-adsorption isotherms of gas mixtures. The theory assumes that the gas-adsorbed phase system is analogous to a vapor-liquid equilibrium state following Raoult’s law [78]. It relies on three major assumptions: (i) the change in the thermodynamic properties of the adsorbent when gas molecules adsorb is negligible compared to the change in those for the adsorbate; (ii) each adsorbed species has access to the same area of the adsorbent surface; (iii) the Gibbs definition applies to the adsorbed phase [77]. IAST provides a solid theoretical foundation for predicting multi-component adsorption isotherms from single-component adsorption isotherms. IAST has been found to reliably predict the co-adsorption isotherms and selectivities of CO_2_/CH_4_ mixtures in MOF adsorbents [20,79].

The computational process of the IAST model is described in detail elsewhere [77,80,81]. In this work, IAST isotherm calculation was performed with the aid of the IAST++ program to assess the separation performance of binary equimolar CO_2_/CH_4_ mixtures at 298 K [81]. Based on the prediction of the IAST co-adsorption isotherms, the equilibrium selectivity of the adsorbent samples α_i/j_ could be determined in the whole range of tested pressure and temperature:

(4) $$\alpha_{i/j}=\;\frac{x_{i,}/y_{i}}{x_{j,}/y_{j}}$$

where x_i_ and x_j_ were the molar fraction of components i and j in the adsorbed phase; and y_i_ and y_j_ were the molar fractions of components i and j in the gas phase.

### 3.11. Water Contact Angle

The water contact angle over the polymer binding agents was measured using the sessile droplet method. Prior to analysis, a flat film of the TPU or PLA sample was prepared using the solvent casting technique. Briefly, 1 g of TPU or PLA was dissolved in DMF or chloroform, respectively. The dissolved polymer was then cast into a glass Petri dish and heated at 383 K overnight to remove the solvent and obtain thermoplastic film samples. Once a flat film sample of thermoplastic was obtained, a distilled water droplet was deposited from a syringe on the surface of the sample. An image of the water droplet was immediately taken by a high-speed digital microscope camera (Amscope (Irvine, CA, USA) MU1000), and the contact angle, θ, between the droplet and the surface of the samples was determined using the ImageJ software (version 1.53t) [82]. The water contact angle can be used as an indicator of surface wettability: when θ < 90°, the sample is considered hydrophilic; when 90° < θ < 120°, the sample is hydrophobic; and when θ > 120°, the sample is super-hydrophobic. Each test was repeated five times, and the reported results were the average of these five measurements.

### 3.12. Water Adsorption Isotherms and Material Aging under Humid Atmosphere

The measurement of H_2_O adsorption isotherms was performed using the manometric equipment, a 3 Flex apparatus from Micromeritics. Water adsorption isotherm data were collected at 298 K from 0 to 2.1 kPa. Prior to each measurement, the material samples were degassed under a dynamic vacuum at 393 K for 12 h, and the sample holder dead volume was measured from helium expansion.

Material aging and stability to moisture were assessed during storage under atmospheric humid conditions by leaving the adsorbent samples inside a room at a controlled temperature of 25 ± 5 °C and at a relative humidity (RH) of 40 ± 5% for a duration of 3 months. The properties of the stored samples were then re-characterized via measurements of the N_2_ adsorption isotherms at 77 K and CO_2_ adsorption isotherms up to 1 bar at 298 K.

## 4. Conclusions

In this work, hydrophobic TPU was used as a binding agent for the shaping of HKUST−1 powder using an extrusion process. The resulting composite was characterized, and its properties were compared with those of synthesized HKUST−1/PLA, obtained after the same shaping process using PLA as a more hydrophilic polymeric binder. The characterization confirms the successful shaping of HKUST−1 powder using TPU as a binder. The HKUST−1/TPU composite exhibited comparable textural properties, thermal stability, and mechanical stability to the HKUST−1/PLA composite. HKUST−1/TPU demonstrated comparable equilibrium adsorption capacities and IAST selectivities as HKUST−1/PLA for CO_2_/CH_4_ separation. The adsorption capacities of both composites were shown to be larger than the HKUST−1 pristine powder. Such an improvement was attributed to the methanol washing effect applied during the composite preparation before their drying. Methanol-washed HKUST−1 powder was characterized by the largest adsorption capacities when compared with pristine powder and both the PLA and TPU composites. Additionally, HKUST−1/TPU had a higher heat of adsorption for CO_2_ than for CH_4_, and these data are not dependent on the amount of gas adsorbed. Finally, as TPU was more hydrophobic than PLA, the use of that polymer as a binding agent contributed to a decrease in the degradation rate of the adsorbent after storage in a humid environment for several months. On the whole, this study demonstrates the feasibility of using TPU as a polymeric binder for the shaping of commercial HKUST−1 powder. The extrusion process results in the production of an adsorbent well-suited to be used in an industrial gas-separation process thanks to its good adsorption performance, mechanical and thermal properties, and stability after a long period of storage in a humid atmosphere.

## Figures and Tables

**Table 1:** BET Surface area, pore volume of HKUST−1, methanol-washed HKUST−1, HKUST−1/TPU and HKUST−1/PLA. Data for HKUST−1 and HKUST−1/PLA was taken from previous work [46].

Sample	S_BET_ (m^2^/g)	Micropore Volume (cm^3^/g)	Total Pore Volume (cm^3^/g)
HKUST−1	1500	0.46	0.65
HKUST−1/TPU	1557	0.50	0.65
HKUST−1/PLA	1528	0.54	0.65
Methanol-washed HKUST−1	1956	0.60	0.79

**Table 2:** Attrition percentage of HKUST−1/TPU, HKUST−1/PLA, conventional adsorbent and MOF extrudates.

Sample	Attrition Loss (% wt)	Reference
HKUST−1/TPU	0.4	This study
HKUST−1/PLA	0.5	[46]
Zeolite 3A	≤0.2	[65]
Zeolite 4A	≤0.2	
Zeolite 5A	≤0.2	
Zeolite 13X	≤0.2	
AC-Norit RZN_1_	0.2	[66]
UiO-66 extrudate	1.4	

**Table 3:** Average value of water contact angle onto the surface of TPU and PLA.

Polymer	Average Contact Angle (°)
PLA	66.9
TPU	90.3

## Data Availability

The original contributions presented in the study are included in the article/Supplementary Material; further inquiries can be directed to the corresponding author/s.

## Supplementary Materials

The following supporting information can be downloaded at https://www.mdpi.com/article/10.3390/molecules29092069/s1, Figure S1: Image of water drop onto the surface of PLA and TPU; Figure S2: N_2_ adsorption isotherm plot for TPU and PLA; Figure S3: N_2_ adsorption isotherms on HKUST−1 and solvent-washed HKUST−1; Figure S4: Normalize CO_2_ adsorption isotherm plot for pristine HKUST−1, methanol-washed HKUST−1 and HKUST−1/PLA composites with different binder fraction; Table S1: Fitting Parameters of the dual-site Langmuir Isotherm Model for the CO_2_ and CH_4_ pure isotherms on HKUST−1, HKUST−1/PLA and HKUST−1/TPU; Table S2: BET Surface area, pore volume of HKUST−1 and the respective solvent-washed HKUST−1.

## References

[1] Pavičić J., Mavar K.N., Brkić V., Simon K. (2022). Biogas and Biomethane Production and Usage: Technology Development, Advantages and Challenges in Europe. Energies.

[2] IEA (2020). Outlook for Biogas and Biomethane: Prospects for Organic Growth. https://www.iea.org/reports/outlook-for-biogas-and-biomethane-prospects-for-organic-growth.

[3] Khan I.U., Othman M.H.D., Hashim H., Matsuura T., Ismail A.F., Rezaei-DashtArzhandi M., Azelee I.W. (2017). Biogas as a renewable energy fuel—A review of biogas upgrading, utilisation and storage. Energy Convers. Manag..

[4] Bharathiraja B., Sudharsana T., Jayamuthunagai J., Praveenkumar R., Chozhavendhan S., Iyyappan J. (2018). Biogas production—A review on composition, fuel properties, feed stock and principles of anaerobic digestion. Renew. Sustain. Energy Rev..

[5] Aghel B., Behaein S., Wongwises S., Shadloo M.S. (2022). A review of recent progress in biogas upgrading: With emphasis on carbon capture. Biomass Bioenergy.

[6] Ahmed S.F., Mofijur M., Tarannum K., Chowdhury A.T., Rafa N., Nuzhat S., Kumar P.S., Vo D.-V.N., Lichtfouse E., Mahlia T.M.I. (2021). Biogas upgrading, economy and utilization: A review. Environ. Chem. Lett..

[7] Shah G., Ahmad E., Pant K., Vijay V. (2021). Comprehending the contemporary state of art in biogas enrichment and CO_2_ capture technologies via swing adsorption. Int. J. Hydrogen Energy.

[8] Jiao L., Seow J.Y.R., Skinner W.S., Wang Z.U., Jiang H.-L. (2019). Metal–organic frameworks: Structures and functional applications. Mater. Today.

[9] Ghazvini M.F., Vahedi M., Nobar S.N., Sabouri F. (2021). Investigation of the MOF adsorbents and the gas adsorptive separation mechanisms. J. Environ. Chem. Eng..

[10] Ullah S., Bustam M.A., Assiri M.A., Al-Sehemi A.G., Gonfa G., Mukhtar A., Kareem F.A.A., Ayoub M., Saqib S., Mellon N.B. (2020). Synthesis and characterization of mesoporous MOF UMCM-1 for CO_2_/CH_4_ adsorption; an experimental, isotherm modeling and thermodynamic study. Microporous Mesoporous Mater..

[11] Salehi S., Anbia M., Razavi F. (2020). Improving CO_2_/CH_4_ and CO_2_/N_2_ adsorptive selectivity of cu-BTC and MOF-derived nanoporous carbon by modification with nitrogen-containing groups. Environ. Prog. Sustain..

[12] Li C.-N., Wang S.-M., Tao Z.-P., Liu L., Xu W.-G., Gu X.-J., Han Z.-B. (2023). Green Synthesis of MOF-801 (Zr/Ce/Hf) for CO_2_/N_2_ and CO_2_/CH_4_ Separation. Inorg. Chem..

[13] Teo H.W.B., Chakraborty A., Kayal S. (2017). Evaluation of CH_4_ and CO_2_ adsorption on HKUST−1 and MIL-101 (Cr) MOFs employing Monte Carlo simulation and comparison with experimental data. Appl. Therm. Eng..

[14] Asadi T., Ehsani M.R., Ribeiro A.M., Loureiro J.M., Rodrigues A.E. (2017). CO_2_/CH_4_ Separation by Adsorption using Nanoporous Metal organic Framework Copper-Benzene-1, 3, 5-tricarboxylate Tablet. Chem. Eng. Technol..

[15] Chong K.C., Lai S.O., Mah S.K., Thiam H.S., Chong W.C., Shuit S.H., Lee S.S., Chong W.E. (2023). A Review of HKUST−1 Metal-Organic Frameworks in Gas Adsorption. Conf. Ser. Earth Environ. Sci..

[16] Yu D., Yazaydin A.O., Lane J.R., Dietzel P.D.C., Snurr R.Q. (2013). A combined experimental and quantum chemical study of CO_2_ adsorption in the metal–organic framework CPO-27 with different metals. Chem. Sci..

[17] Millward A.R., Yaghi O.M. (2005). Metal−organic frameworks with exceptionally high capacity for storage of carbon dioxide at room temperature. J. Am. Chem. Soc..

[18] Wu H., Zhou W., Yildirim T. (2009). High-capacity methane storage in metal−organic frameworks M2 (dhtp): The important role of open metal sites. J. Am. Chem. Soc..

[19] Ye S., Jiang X., Ruan L.-W., Liu B., Wang Y.-M., Zhu J.-F., Qiu L.-G. (2013). Post-combustion CO_2_ capture with the HKUST−1 and MIL-101 (Cr) metal–organic frameworks: Adsorption, separation and regeneration investigations. Microporous Mesoporous Mater..

[20] Hamon L., Jolimaître E., Pirngruber G.D. (2010). CO_2_ and CH_4_ separation by adsorption using Cu-BTC metal−organic framework. Ind. Eng. Chem. Res.

[21] Xian S., Peng J., Zhang Z., Xia Q., Wang H., Li Z. (2015). Highly enhanced and weakened adsorption properties of two MOFs by water vapor for separation of CO_2_/CH_4_ and CO_2_/N_2_ binary mixtures. Chem. Eng. J..

[22] Chanut N., Wiersum A.D., Lee U.-H., Hwang Y.K., Ragon F., Chevreau H., Bourrelly S., Kuchta B., Chang J.-S., Serre C. (2016). Observing the effects of shaping on gas adsorption in metal-organic frameworks. Eur. J. Inorg. Chem..

[23] Bourrelly S., Llewellyn P.L., Serre C., Millange F., Loiseau T., Férey G. (2005). Different adsorption behaviors of methane and carbon dioxide in the isotypic nanoporous metal terephthalates MIL-53 and MIL-47. J. Am. Chem. Soc.

[24] Kayal S., Chakraborty A. (2018). Activated carbon (type Maxsorb-III) and MIL-101 (Cr) metal organic framework based composite adsorbent for higher CH_4_ storage and CO_2_ capture. Chem. Eng. J..

[25] Yuan D., Zhao D., Sun D., Zhou H.-C. (2010). An isoreticular series of metal–organic frameworks with dendritic hexacarboxylate ligands and exceptionally high gas-uptake capacity. Angew. Chem..

[26] Lv D., Shi R., Chen Y., Chen Y., Wu H., Zhou X., Xi H., Li Z., Xia Q. (2018). Selective adsorptive separation of CO_2_/CH_4_ and CO_2_/N_2_ by a water resistant zirconium–porphyrin metal–organic framework. Ind. Eng. Chem. Res..

[27] Awadallah-F A., Hillman F., Al-Muhtaseb S.A., Jeong H.K. (2019). Adsorption equilibrium and kinetics of nitrogen, methane and carbon dioxide gases onto ZIF-8, Cu10%/ZIF-8, and Cu30%/ZIF-8. Ind. Eng. Chem. Res..

[28] Rocha L.A., Andreassen K.A., Grande C.A. (2017). Separation of CO_2_/CH_4_ using carbon molecular sieve (CMS) at low and high pressure. Chem. Eng. Sci..

[29] Cavenati S., Grande C.A., Rodrigues A.E. (2004). Adsorption equilibrium of methane, carbon dioxide, and nitrogen on zeolite 13X at high pressures. J. Chem. Eng. Data.

[30] Ren J., Dyosiba X., Musyoka N.M., Langmi H.W., Mathe M., Liao S. (2017). Review on the current practices and efforts towards pilot-scale production of metal-organic frameworks (MOFs). Coord. Chem. Rev..

[31] Golmakani A., Nabavi S.A., Wadi B., Manovic V. (2022). Advances, challenges, and perspectives of biogas cleaning, upgrading, and utilisation. Fuel.

[32] Álvarez J.R., Sánchez-González E., Pérez E., Schneider-Revueltas E., Martínez A., Tejeda-Cruz A., Islas-Jácome A., González-Zamora E., Ibarra I.A. (2017). Structure stability of HKUST−1 towards water and ethanol and their effect on its CO_2_ capture properties. Dalton Trans..

[33] Al-Janabi N., Hill P., Torrente-Murciano L., Garforth A., Gorgojo P., Siperstein F., Fan X. (2015). Mapping the Cu-BTC metal–organic framework (HKUST−1) stability envelope in the presence of water vapour for CO_2_ adsorption from flue gases. Chem. Eng. J..

[34] Ediati R., Dewi S.K., Hasan M.R., Kahardina M., Murwani I.K., Nadjib M. (2019). Mesoporous HKUST−1 synthesized using solvothermal method. Rasayan J. Chem..

[35] Morales E.M.C., Méndez-Rojas M.A., Torres-Martínez L.M., Garay-Rodríguez L.F., López I., Uflyand I.E., Kharisov B.I. (2021). Ultrafast synthesis of HKUST−1 nanoparticles by solvothermal method: Properties and possible applications. Polyhedron.

[36] Nobar S.N. (2018). Cu-BTC synthesis, characterization and preparation for adsorption studies. Mater. Chem. Phys..

[37] Guo L., Du J., Li C., He G., Xiao Y. (2021). Facile synthesis of hierarchical micro-mesoporous HKUST−1 by a mixed-linker defect strategy for enhanced adsorptive removal of benzothiophene from fuel. Fuel.

[38] Armstrong M., Sirous P., Shan B., Wang R., Zhong C., Liu J., Mu B. (2018). Prolonged HKUST−1 functionality under extreme hydrothermal conditions by electrospinning polystyrene fibers as a new coating method. Microporous Mesoporous Mater..

[39] Vehrenberg J., Vepsäläinen M., Macedo D.S., Rubio-Martinez M., Webster N.A.S., Wessling M. (2020). Steady-state electrochemical synthesis of HKUST−1 with polarity reversal. Microporous Mesoporous Mater..

[40] Vepsäläinen M., Macedo D.S., Gong H., Rubio-Martinez M., Bayatsarmadi B., He B. (2021). Electrosynthesis of HKUST−1 with flow-reactor post-processing. Appl. Sci..

[41] Liu P., Zhao T., Cai K., Chen P., Liu F., Tao D.-J. (2022). Rapid mechanochemical construction of HKUST−1 with enhancing water stability by hybrid ligands assembly strategy for efficient adsorption of SF6. Chem. Eng. J..

[42] Stolar T., Batzdorf L., Lukin S., Žilić D., Motillo C., Friščić T., Emmerling F., Halasz I., Užarević K. (2017). In situ monitoring of the mechanosynthesis of the archetypal metal–organic framework HKUST−1: Effect of liquid additives on the milling reactivity. Inorg. Chem..

[43] Ntouros V., Kousis I., Pisello A.L., Assimakopoulos M.N. (2022). Binding Materials for MOF Monolith Shaping Processes: A Review towards Real Life Application. Energies.

[44] Cousin-Saint-Remi J., Finoulst A.-L., Jabbour C., Baron G.V., Denayer J.F.M. (2020). Selection of binder recipes for the formulation of MOFs into resistant pellets for molecular separations by fixed-bed adsorption. Microporous Mesoporous Mater..

[45] Hastürk E., Höfert S.-P., Topalli B., Schlüsener C., Janiak C. (2020). Shaping of MOFs via freeze-casting method with hydrophilic polymers and their effect on textural properties. Microporous Mesoporous Mater..

[46] Rozaini M.T., Grekov D.I., Bustam M.A., Pré P. (2023). Shaping of HKUST−1 via Extrusion for the Separation of CO_2_/CH_4_ in Biogas. Separations.

[47] Yu W., Sun L., Li M., Li M., Lei W., Wei C. (2023). FDM 3D printing and properties of PBS/PLA blends. Polymers.

[48] Rajakaruna R.A., Subeshan B., Asmatulu E. (2022). Fabrication of hydrophobic PLA filaments for additive manufacturing. J. Mater. Sci..

[49] Nofar M., Mohammadi M., Carreau P.J. (2020). Effect of TPU hard segment content on the rheological and mechanical properties of PLA/TPU blends. J. Appl. Polym. Sci.

[50] Lis-Bartos A., Smieszek A., Frańczyk K., Marycz K. (2018). Fabrication, characterization, and cytotoxicity of thermoplastic polyurethane/poly (lactic acid) material using human adipose derived mesenchymal stromal stem cells (hASCs). Polymers.

[51] Oliaei E., Kaffashi B., Davoodi S. (2016). Investigation of structure and mechanical properties of toughened poly (l-lactide)/thermoplastic poly (ester urethane) blends. J. Appl. Polym. Sci..

[52] Muñoz-Chilito J., Lara-Ramos J.A., Marín L., Machuca-Martínez F., Correa-Aguirre J.P., Hidalgo-Salazar M.A., García-Navarro S., Roca-Blay L., Rodríguez L.A., Mosquera-Vargas E. (2023). Morphological Electrical and Hardness Characterization of Carbon Nanotube-Reinforced Thermoplastic Polyurethane (TPU) Nanocomposite Plates. Molecules.

[53] Kumar S., Gupta T.K., Varadarajan K.M. (2019). Strong, stretchable and ultrasensitive MWCNT/TPU nanocomposites for piezoresistive strain sensing. Compos. Part B.

[54] Dai X., Cao Y., Shi X., Wang X. (2016). Non-isothermal crystallization kinetics, thermal degradation behavior and mechanical properties of poly (lactic acid)/MOF composites prepared by melt-blending methods. RSC Adv..

[55] Gregor-Svetec D., Leskovšek M., Leskovar B., Elesini U.S., Vrabič-Brodnjak U. (2021). Analysis of PLA composite filaments reinforced with lignin and polymerised-lignin-treated NFC. Polymers.

[56] Yang T., Hu J., Wang P., Edeleva M., Cardon L., Zhang J. (2023). Two-step approach based on fused filament fabrication for high performance graphene/thermoplastic polyurethane composite with segregated structure. Compos. Part A Appl. Sci. Manuf..

[57] Bhoria N., Basina G., Pokhrel J., Reddy K.S.K., Anastasiou S., Balasubramanian V.V., AlWahedi Y.F., Karanikolos G.N. (2020). Functionalization effects on HKUST−1 and HKUST−1/graphene oxide hybrid adsorbents for hydrogen sulfide removal. J. Hazard. Mater..

[58] Cortés-Súarez J., Celis-Arias V., Beltra H.I., Tejeda-Cruz A., Ibarra I.A., Romero-Ibarra E., Sánchez-González E., Loera-Serna S. (2019). Synthesis and characterization of an SWCNT@ HKUST−1 composite: Enhancing the CO_2_ adsorption properties of HKUST−1. ACS Omega.

[59] Cychosz K.A., Guillet-Nicolas R., García-Martínez J., Thommes M. (2017). Recent advances in the textural characterization of hierarchically structured nanoporous materials. Chem. Soc. Rev..

[60] Kanbur Y., Tayfun U. (2018). Investigating mechanical, thermal, and flammability properties of thermoplastic polyurethane/carbon nanotube composites. J. Thermoplast. Compos. Mater..

[61] Wu W., Huang W., Tong Y., Huang J., Wu J., Cao X., Zhang Q., Yu B., Li R.K.Y. (2023). Self-assembled double core-shell structured zeolitic imidazole framework-8 as an effective flame retardant and smoke suppression agent for thermoplastic polyurethane. Appl. Surf. Sci..

[62] Evans K.A., Kennedy Z.C., Arey B.W., Christ J.F., Schaef H.T., Nune S.K., Erikson R.L. (2018). Chemically active, porous 3D-printed thermoplastic composites. ACS Appl. Mater. Interfaces.

[63] Zhang J., Li Z., Qi X., Zhang W., Wang D.-Y. (2020). Size tailored bimetallic metal-organic framework (MOF) on graphene oxide with sandwich-like structure as functional nano-hybrids for improving fire safety of epoxy. Compos. Part B.

[64] (2020). Standard Test Method for Attrition and Abrasion of Catalysts and Catalyst Carriers.

[65] Shah B.B., Kundu T., Zhao D. (2019). Mechanical properties of shaped metal-organic frameworks. Top. Curr. Chem..

[66] Khabzina Y., Dhainaut J., Ahlhelm M., Richter H.-J., Reinsch H., Stock N., Farrusseng D. (2018). Synthesis and shaping scale-up study of functionalized UiO-66 MOF for ammonia air purification filters. Ind. Eng. Chem. Res..

[67] Supronowicz B., Mavrandonakis A., Heine T. (2013). Interaction of small gases with the unsaturated metal centers of the HKUST−1 metal organic framework. J. Phys. Chem. C.

[68] García-Pérez E., Gascón J., Morales-Flórez V., Castillo J.M., Kapteijn F., Calero S. (2009). Identification of adsorption sites in Cu-BTC by experimentation and molecular simulation. Langmuir.

[69] Chae Y.S., Park S., Kang D.W., Kim D.W., Kang M., Choi D.S., Choe J.H., Hong C.S. (2022). Moisture-tolerant diamine-appended metal–organic framework composites for effective indoor CO_2_ capture through facile spray coating. Chem. Eng. J..

[70] DeCoste J.B., Denny M.S., Peterson G.W., Mahle J.J., Cohen S.M. (2016). Enhanced aging properties of HKUST−1 in hydrophobic mixed-matrix membranes for ammonia adsorption. Schem. Sci..

[71] Park J., Chae Y.S., Kang D.W., Kang M., Choe J.H., Kim S., Kim J.Y., Jeong Y.W., Hong C.S. (2021). Shaping of a metal–organic framework–polymer composite and its CO_2_ adsorption performances from humid indoor air. ACS Appl. Mater. Interfaces.

[72] Xue W., Zhang Z., Huang H., Zhong C., Mei D. (2019). Theoretical insights into the initial hydrolytic breakdown of HKUST−1. J. Phys. Chem. C.

[73] Todaro M., Alessi A., Sciortino L., Agnello S., Cannas M., Gelardi F.M., Buscarino G. (2016). Investigation by Raman Spectroscopy of the Decomposition Process of HKUST−1 upon Exposure to Air. J. Spectrosc..

[74] Rouquerol J., Rouquerol F., Llewellyn P., Maurin G., Sing K. (2013). Adsorption by Powders and Porous Solids: Principles, Methodology and Applications.

[75] Sircar S., Mohr R., Ristic C., Rao M.B. (1999). Isosteric heat of adsorption: Theory and experiment. J. Phys. Chem. B.

[76] Tian Y., Wu J. (2017). Differential heat of adsorption and isosteres. Langmuir.

[77] Myers A.L., Prausnitz J.M. (1965). Thermodynamics of mixed-gas adsorption. AIChE J..

[78] Walton K.S., Sholl D.S. (2015). Predicting multicomponent adsorption: 50 years of the ideal adsorbed solution theory. AIChE J..

[79] Heymans N., SVaesen, De Weireld G. (2012). A complete procedure for acidic gas separation by adsorption on MIL-53 (Al). Microporous Mesoporous Mater..

[80] Simon C.M., Smit B., Haranczyk M. (2016). pyIAST: Ideal adsorbed solution theory (IAST) Python package. Comput. Phys. Commun..

[81] Lee S., Lee J.H., Kim J. (2018). User-friendly graphical user interface software for ideal adsorbed solution theory calculations. Korean J. Chem. Eng..

[82] Rasband W.S. “ImageJ”. U.S. National Institutes of Health, Bethesda, Maryland. https://imagej.nih.gov/ij/.

