# Supplementary material for: Low-Hydrophilic HKUST−1/Polymer Extrudates for the PSA Separation of CO2/CH4
Source: Molecules. 2024 Apr 30;29(9):2069. doi: 10.3390/molecules29092069 (PMC11085341; doi:10.3390/molecules29092069)
Supplement: Supplementary file 1 [file molecules-29-02069-s001.zip › molecules-2963191-supplementary.pdf]

Low-Hydrophilic HKUST−1/Polymer Extrudates for the PSA Separation of CO_2/CH_4

Muhamad Tahriri Rozaini [1,2], Denys I. Grekov [2], Mohamad Azmi Bustam [1,*] and Pascaline Pré [2,*]

[1] Centre of Research in Ionic Liquids, CORIL, Chemical Engineering Department, Universiti Teknologi Petronas, Bandar Seri Iskandar 32610 Perak, Malaysia; muhamad_21000026@utp.edu.my or muhamad-tahriri.bin-rozaini@imt-atlantique.fr
[2] GEnie des Procédés Environnement-Agroalimentaire (GEPEA) UMR-CNRS 6144, Department of Energy Systems and Environment, IMT Atlantique, 44300 Nantes, France; denys.grekov@imt-atlantique.fr
* Correspondence: azmibustam@utp.edu.my (M.A.B.); pascaline.pre@imt-atlantique.fr (P.P.)

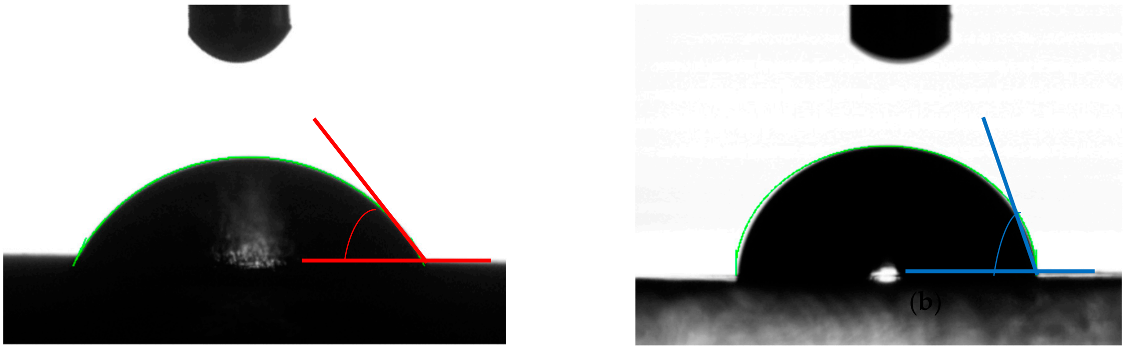

Figure S1: Image of water drop onto the surface of PLA (left) and TPU (right).

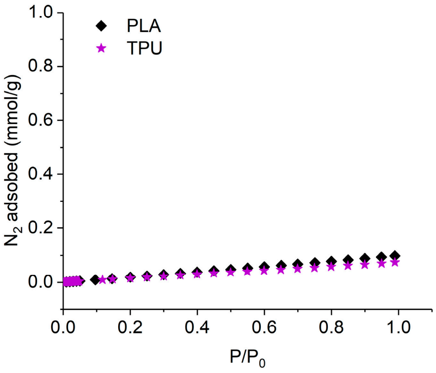

Figure S2. N_2 adsorption isotherm plot for TPU and PLA. Data for PLA was taken from previous study [1]

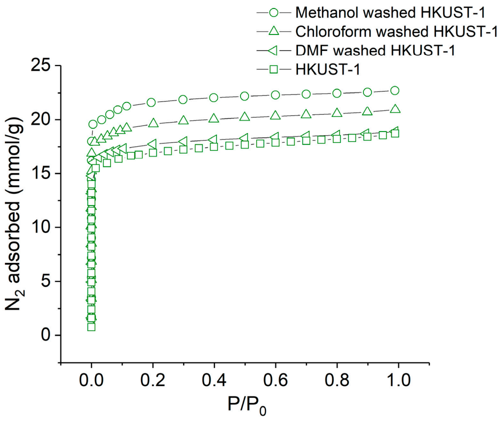

Figure S3. N_2 adsorption isotherms on HKUST−1 and solvent-washed HKUST−1.

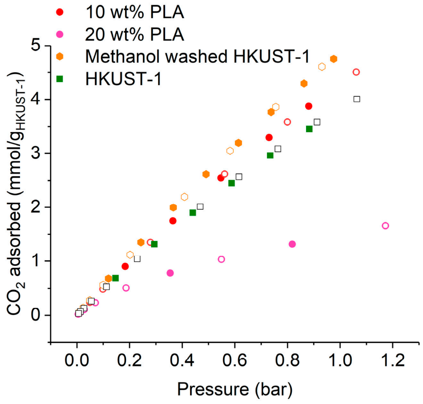

Figure S4: Normalize CO_2 adsorption isotherm plot for pristine HKUST−1, methanol washed HKUST−1 and HKUST−1/PLA composites with different binder fraction.

Table S1: Fitting Parameters of the Dual-site Langmuir Isotherm Model for the CO_2 and CH_4 pure isotherms on HKUST−1, HKUST−1/PLA and HKUST−1/TPU.

		HKUST−1						HKUST−1/PLA						HKUST−1/TPU					
		CO_2			CH_4			CO_2			CH_4			CO_2			CH_4		
Isotherm model	Fitted parameters	273 K	298 K	323 K	273 K	298 K	323 K	273 K	298 K	323 K	273 K	298 K	323 K	273 K	298 K	323 K	273 K	298 K	323 K
Dual-Site Langmuir	R^2	0.998	0.999	0.999	0.998	0.997	0.998	0.998	0.998	0.999	0.999	0.999	0.998	0.998	0.997	0.999	0.999	0.996	0.996
	q_{s1} (mol/kg)	12.23			9.81			14.61			16.07			14.50			15.30		
	q_{s2} (mol/kg)	2.09			2.09			2.09			2.09			2.09			2.09		
	k_{a1} (bar^{-1})	116.76			1.83			48.14			0.67			44.23			1.10		
	k_{a2} (K^{-1})	3066.95			1762.24			2685.58			1418.62			2614.42			1724.25		
	k_{b1} (bar^{-1})	60.29			60.28			60.29			60.28			60.29			60.28		
	k_{b2} (K^{-1})	1404.2			36328.4			14404.1			36328.42			14404.5			36328.42		

Table S2. BET Surface area, pore volume of HKUST−1 and the respective solvent-washed HKUST−1.

**Sample**	**S_{BET} (m^2/g)**	**Micropore volume (cm^3/g)**	**Total pore volume (cm^3/g)**
Pristine HKUST−1	1500	0.46	0.65
DMF washed HKUST−1	1616	0.46	0.65
Chloroform washed HKUST−1	1762	0.51	0.72
Methanol washed HKUST−1	1956	0.60	0.79

References

1. Rozaini, Muhamad Tahriri, Denys I. Grekov, Mohamad Azmi Bustam, and Pascaline Pré, "Shaping of HKUST−1 via Extrusion for the Separation of CO2/CH4 in Biogas," Separations , vol. 10, no. 9, p. 487, 2023.
